# Palm Vein Verification Using Multiple Features and Locality Preserving Projections

**DOI:** 10.1155/2014/246083

**Published:** 2014-02-17

**Authors:** Ali Mohsin Al-juboori, Wei Bu, Xiangqian Wu, Qiushi Zhao

**Affiliations:** ^1^School of Computer Science and Technology, Harbin Institute of Technology, Harbin 150001, China; ^2^College of Computer Science and Mathematics, University of Al-Qadisiyah, Iraq; ^3^Department of New Media Technology and Arts, Harbin Institute of Technology, Harbin 150001, China

## Abstract

Biometrics is defined as identifying people by their physiological characteristic, such as iris pattern, fingerprint, and face, or by some aspects of their behavior, such as voice, signature, and gesture. Considerable attention has been drawn on these issues during the last several decades. And many biometric systems for commercial applications have been successfully developed. Recently, the vein pattern biometric becomes increasingly attractive for its uniqueness, stability, and noninvasiveness. A vein pattern is the physical distribution structure of the blood vessels underneath a person's skin. The palm vein pattern is very ganglion and it shows a huge number of vessels. The attitude of the palm vein vessels stays in the same location for the whole life and its pattern is definitely unique. In our work, the matching filter method is proposed for the palm vein image enhancement. New palm vein features extraction methods, global feature extracted based on wavelet coefficients and locality preserving projections (WLPP), and local feature based on local binary pattern variance and locality preserving projections (LBPV_LPP) have been proposed. Finally, the nearest neighbour matching method has been proposed that verified the test palm vein images. The experimental result shows that the EER to the proposed method is 0.1378%.

## 1. Introduction

The hand vein pattern is here regarded as a biometric feature. Biometrics is the automated measurement of physiological or behavioural characteristics to determine or verify identity. Physical characteristics of the human body are biological and behavioural properties. The hand vein pattern is used as a biometric features, in this case a biological characteristic of the person can be detected and from which distinguishing. The biometric features can be extracted for the purpose of automated recognition of persons. Verification is affirmation by test and provision of objective proof that specified requirements have been fulfilled. Verification in a biometric application is the outcome true or false on a claim about the similarity of a biometric reference and a recognition biometric sample by making a comparison, a one-to-one match. Biometric authentication is often used as a synonym for verification, but formally this is deprecated. Biometric identification is the outcome of a biometric system function that implements a one-to-many search to obtain a filter list [1]. As security requirements increase, biometric techniques, including face, fingerprint, iris, voice, and vein recognitions, have been widely used for personal identification. Biometrics has been applied to building access control, immigration control, and user authentication for financial transactions. Although vein recognition has not been as widely adopted as fingerprint, face, and iris recognition, it has some advantages. Since vein patterns exist inside the skin, it is very difficult to steal them. The vein patterns are not easily altered by other factors such as dry or wet skin. Palm vein recognition is one form of vein pattern recognition, which identifies a user based on palm vein patterns. Palm vein recognition, investigated in previous studies, uses vein patterns in the palm for personal identification. Hand and palm vein recognitions, however, have a disadvantage in that the dimensions of the device are inevitably large because the vascular features from the whole hand are extracted while capturing a vein image [2].

There are many studies on the vein pattern recognition. In [2] the researchers proposed a method for finger vein patterns using a weighted local binary pattern (LBP) and support vector machine (SVM). Without using any preprocessing methods, the LBP codes are extracted. And classifying the LBP codes that extracted into three categories depends on the SVM classifier: large amount (LA), medium amount (MA), and small amount (SA) of finger vein patterns. According to these areas, different weights are given to the extracted LBP code. The disadvantage of the work, the experimental, is tested on small database contains only 960 finger vein images. In [3] the researchers worked on PolyU multispectral palmprint database. The researchers combined the palmprint and palm vein for their proposed system. The matching filter method is used to extract the vein. The EER to the system is 0.3091% based on palm vein only, while they used fused multimodal (palmprint and palm vein) features to evaluate the system. In [4] the researchers extract multiscale LBP features of hand vein images. The hand vein image is decomposed with two-level wavelet and gets eight subband coefficient matrices and computes the weight of each subband; the recognition accuracy of each subband is calculated by original LBP based on Euclidean distance. The experimental results reported the EER value to their proposed method is 2.067%. In [5] finger vein recognition has been identified using local binary pattern variance (LBPV). Global matching method is used to get more speeding and to decrease feature dimensions using distance measurement. The classification rate of this method is tested using support vector machine (SVM). In [6] the researcher presents a multimodal personal identification system using palmprint and palm vein images with their fusion applied at the image level. The palmprint and palm vein images are fused by a new edge-preserving and contrast-enhancing wavelet fusion method in which the modified multiscale edges of the palmprint and palm vein images are combined. A palm representation, called “Laplacianpalm” feature, is extracted from the fused images by the locality preserving projections (LPP). In [7] the researcher presents two new palm vein representations, using Hessian phase information from the enhanced vascular patterns in the normalized images and secondly from the orientation encoding of palm vein line-like patterns using localized Radon transform. In [8] the researchers consider the palm vein as a texture and apply feature extraction based on texture vein for person authentication. The feature extracted from the palm vein is based on 2D Gabor filter. Then a directional code technique is proposed to represent the palm vein features called vein code. The matching between two vein codes is computed by normalized Hamming distance. In [9] the researchers worked on PolyU multispectral palmprint database. They used a multiscale curvelet transform as a feature extraction and used a subset from the features for matching using Hamming distance. The lowest EER to their system is 0.66% which is got when selected (40%) from the features set. In [10] the vein feature representation method is called orientation of local binary pattern (OLBP) which is an extension of local binary pattern (LBP). Based on OLBP feature representation, construct a hand vein recognition system employing multiple hand vein patterns that include palm vein, dorsal vein, and three finger veins (index, middle, and ring finger). Vein images are enhanced using Gaussian matched filter and extracted OLBP features and matched. Finally, the matching scores are fused using support vector machine (SVM) to make a decision. In the previous work [11], we implemented a palm vein verification system. The features extraction depends on the Gabor filter with 8 scales and 8 directions. A new dimension reduction method is proposed called Fisher Vein. The matching step is depending on the Nearest Neighbour method. The EER to the system is 0.2335%.

**Figure 1:**
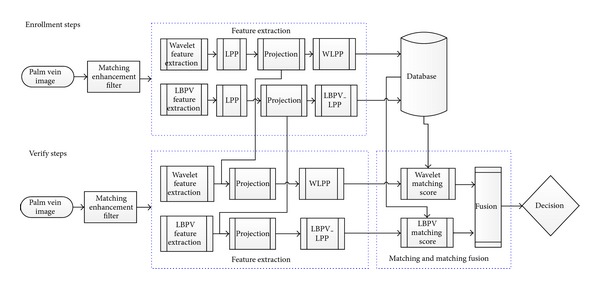
System flowchart.

This paper proposes a multiple features extraction based on global and locate features and merging with locality preserving projections (LPP). The global features extracted using wavelet transform coefficients combine with locality preserving projections and create feature vector called wavelet locality preserving projections (WLPP) and the local binary pattern variance (LBPV) represents the locale features for the palm vein image combined with locality preserving projections and create feature vector called local binary pattern variance-locality preserving projections (LBPV-LPP). Based on the proposed palm vein features representation methods, a palm vein authentication system is constructed. The nearest neighbour method is proposed to match the test palm vein images. The system flowchart is shown in Figure 1. First of all, the palm vein image is enhanced using matching filter and then the features are extracted (WLPP and LBPV-LPP). After similarity measure for each feature, fusion technique is applied to fuse all the matching scores to obtain a final decision.

The rest of this paper is organized as follows.  Section 2 describes the preprocessing.  Section 3 describes the feature extraction methods.  Section 4 describes the matching and matching score fusion strategy used in this work. Finally, the experimental result and conclusions are drawn in Section 5.

## 2. Preprocessing 

It is observed that the cross-sections of palm veins are similar to Gaussian functions. Based on this observation, the matched filters [14, 15], which are widely used in retinal vessel extraction, can be a good technique to extract these palm veins. The matched filters are Gaussian shaped filters along angle *∅*.

Consider

**Figure 2:**
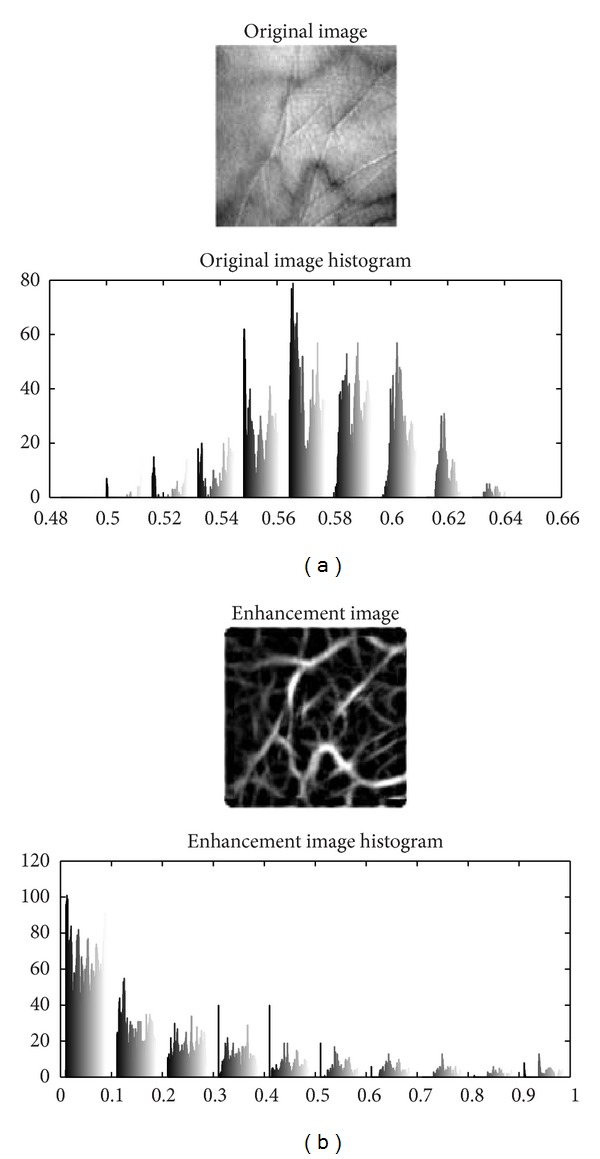
Palm vein image enhancements.

(1) gθσ(x,y)=−exp⁡⁡(−x′2σ2)−m,   for  |x′|≤3σ, |y′|≤L2,

where *x*′ = *x*cos⁡*θ* + *y*sin*θ*, *y*′ = −*x*sin*θ* + *y*cos⁡*θ*, *σ* is the standard deviation of Gaussian, *m* is the mean value of the filter, and *L* is the length of the filter in *y* direction which is set empirically. In order to suppress the background pixels, the filter is designed as a zero-sum. For one *σ*, six different angle filters (*θj* = *jπ*/6, where *j* = {0, 1, 2, 3,4, 5}) are applied for each pixel, and the maximal response among these six directions is kept as the final response for the given scale:

(2) $$\begin{array}{c} R_{F}^{\sigma }=\max\left(R_{\theta j}^{\sigma }\left(x,y\right)\right),\;j=\left\{0,1,2,3,4,5\right\}\\ R_{\theta j}^{\sigma }\left(x,y\right)=g_{\theta j}^{\sigma }*f\left(x,y\right), \end{array}$$

where *f*(*x*, *y*) is the original image and ∗ denotes the convolution operation [3, 14, 15]. The value of *σ* is set empirical.Figure 2 shows the result of palm vein enhancement.

## 3. Feature Extraction

The feature extraction step aims to extract the actual features of the palm vein pattern from the image and then used it for the matching. If the image is an enrolled sample, the features are saved in a training database for later matching. Once the features are extracted, they are compared with the ones in the database and based on that comparison a decision is taken. In the propose work, new palm vein features extraction methods are proposed based on wavelet locality preserving projections (WLPP) features and local binary pattern variance-locality preserving projections (LBPV_LPP) features.

### 3.1. Feature Extraction Based on 2D Wavelet Transform

**Figure 3:**
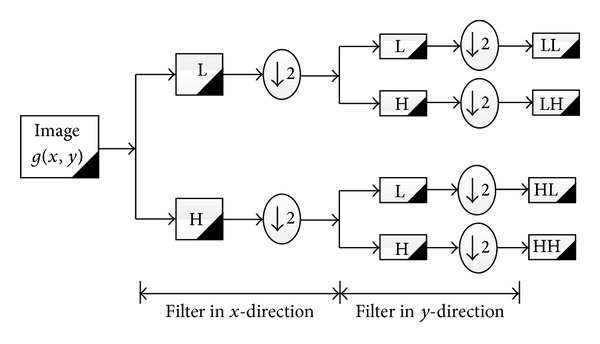
Decomposition algorithm of 2D wavelet transform [12].

**Figure 4:**
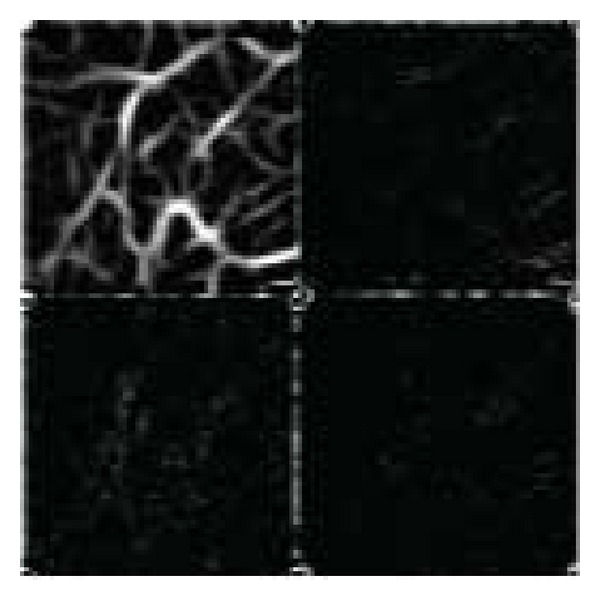
db2 result.

A reliable and robust feature extraction is important for pattern recognition tasks. Recently, a lot of research work has been directed towards using wavelet based features. The discrete wavelet transform (DWT) has a good time and frequency resolution and hence it can be used for extracting the localized contributions of the signal of interest. The mathematical tool for a hierarchically decomposing is the wavelet transform. The wavelet function described the coarse overall shape and details that range from wide to close. The wavelet function an efficient technique for representing the image, surface and curve. The discrete wavelet transform (DWT) is a very popular tool for the analysis of nonstationary signals. The idea of it is to represent a signal as a series of approximations: low-pass version corresponds to the signal, and high-pass version corresponds to details. This is done at different resolutions. It is almost equivalent to filtering the signal with a bank of band pass filters whose impulse responses are all roughly given by scaled versions of a mother wavelet [4, 16, 17]. Multidimensional wavelets (especially the two dimensional 2D wavelets) can be treated as two 1D wavelet transforms (one 1D wavelet transform along the row direction and the other 1D wavelet transform along the column direction). Thus, the 2D wavelet transform can be computed in cascade by filtering the rows and columns of images with 1D filters; Figure 3 shows a one-octave decomposition of an image into four components: low-pass rows, low-pass columns (LL); low-pass rows, high-pass columns (LH); high-pass rows, low-pass columns (HL); high-pass rows, high-pass columns (HH). The coefficients obtained by applying the 2D wavelet transform on an image are called the subimages of wavelet transform [12].  Figure 4 shows the result of db2 on the enhanced palm vein image.

### 3.2. Feature Extraction Based on Local Binary Pattern Variance (LBPV)

**Figure 5:**
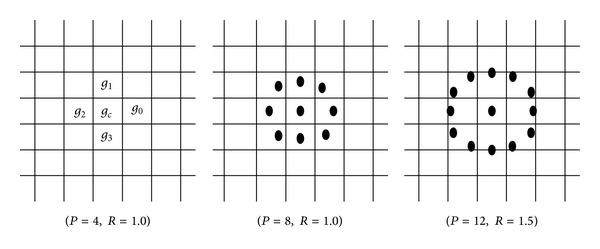
LBP neighbours sets for different (*P*, *R*) [5, 13].

The local binary pattern variance (LBPV) computes the variance (VAR) from a local region and accumulates it into the local binary pattern bin. This can be considered as the integral projection along the VAR coordinate. The spatial structure of the local image texture can be represented by LBP. The pattern number is computed by comparing the center pixel value with those of its neighbours as shown in Figure 5. The LBP is computed by

(3) $$\begin{array}{c} {\text{LBP}}_{P,R}=\sum_{p=0}^{P-1}{s\left(g_{P}-g_{c}\right)2}^{P},\\ s\left(x\right)=\{\begin{array}{cc} 1, & x\geq 0,\\ 0, & x<0, \end{array} \end{array}$$

where *g*_*c*_ is the gray value of the central pixel, *g*_*P*_ is the value of its neighbours, *P* is the number of neighbours, and *R* is the radius of the neighbourhoods. Suppose that the coordinates of *g*_*c*_ are (0,0); then the coordinates of*g*_*P*_ are given by (−*R*sin(2*πp*/*P*), *R*cos⁡(2*πp*/*P*)).  Figure 4 shows examples of circularly symmetric neighbour sets for different configurations of (*P*, *R*). By using the interpolation the gray value of the neighbors can be estimated [5, 13, 18].

**Figure 6:**
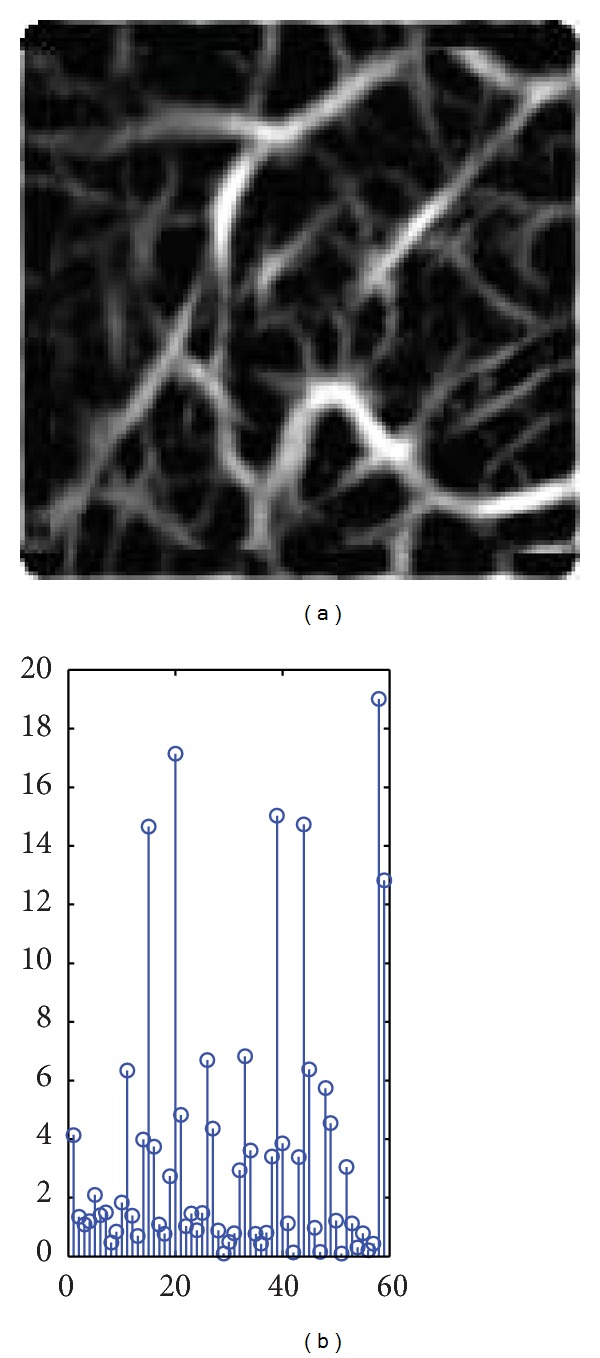
Palm vein image and LBPV histogram.

The local spatial pattern and local contrast can be described briefly by LBP_*P,R*_/VAR_*P,R*_. VAR_*P,R*_ has a continuous value that has to be quantized which can be done by first calculating feature distributions from all training images to get a total distribution and then, to guarantee the highest quantization resolution, some threshold values are computed to partition the total distribution into *N* bins with an equal number of entries. The threshold values are used to quantize the VAR of the test images. There are three particular limitations to this quantization method as stated by [13]. The LBPV proposes a solution to these problems of the descriptor. The information of the variance VAR_*P,R*_ is not involved in the calculation of the LBP histogram. The histogram operation allocates the same weight to each LBP pattern independent of the LBP variance of the local region. The LBPV is given as

(4) $$\begin{array}{c} {\text{LBPV}}_{PR}\left(K\right)=\sum_{i=1}^{N}\sum_{j=1}^{M}W\left({\text{LBP}}_{PR}\left(i,j\right),k\right),\;k\in \left[0,K\right]\\ W\left({\text{LBP}}_{PR}\left(i,j\right),k\right)=\{\begin{array}{cc} {\text{VAR}}_{PR}\left(i,j\right), & {\text{LBP}}_{PR}\left(i,j\right)=k,\\ 0, & \text{otherwise}, \end{array} \end{array}$$

where *K* is the maximal LBP pattern value. It is observed that LBPV is a simplified descriptor whose feature size is small that can be utilized in many applications. It is also training-free and it does not need quantization.  Figure 6 shows the result of the LBPV histogram for the palm vein image.

### 3.3. Locality Preserving Projections

In most computer vision and pattern recognition problems, the large number of sensory inputs, such as images and videos, is computationally challenging to analyze. In such cases it is desirable to reduce the dimensionality of the data while preserving the original information in the data distribution, allowing for more efficient learning and inference. If the variance of the multivariate data is faithfully represented as a set of parameters, the data can be considered as a set of geometrically related points lying on a smooth low-dimensional manifold. The fundamental issue in dimensionality reduction is how to model the geometry structure of the manifold and produce a faithful embedding for data projection. Linear dimensionality reduction (LDR) techniques have been increasingly important in pattern recognition since they permit a relatively simple mapping of data onto a lower dimensional subspace, leading to simple and computationally efficient classification strategies. The main advantage of the linear methods over the nonlinear ones is that the embedding function of the linear techniques is defined everywhere in the input space, while for nonlinear embedding techniques, it is only defined for a set of data samples [19]. Locality preserving projection (LPP) is a manifold learning method widely used in pattern recognition and computer vision. LPP is also well known as a linear graph embedding method. When LPP transforms different samples into new representations using the same linear transform, it tries to preserve the local structure of the samples, that is, the neighbor relationship between samples so that samples that were originally in close proximity in the original space remain so in the new space [20].

The locality preserving projections (LPP) are linear projective maps that arise by solving a variational problem that optimally preserves the neighborhood structure of the data set. LPP should be seen as an alternative to principal component analysis (PCA) which is a classical linear technique that projects the data along the directions of maximal variance. When the high-dimensional data lies on a low-dimensional manifold embedded in the ambient space, the locality preserving projections are obtained by finding the optimal linear approximations to the eigenfunctions of the Laplace Beltrami operator on the manifold. The LPP builds a graph incorporating neighborhood information of the data set. Using the notion of the Laplacian of the graph, compute a transformation matrix which maps the data points to a subspace. This linear transformation optimally preserves local neighborhood information in a certain sense. The representation map generated by the algorithm may be viewed as a linear discrete approximation to a continuous map that naturally arises from the geometry of the manifold locality preserving projection (LPP) which is a linear approximation of the nonlinear Laplacian Eigenmap. In the proposed work, the algorithm procedure that is used as show in [21–23].

(1) *Constructing the Adjacency Graph*. Let *G* denote a graph with *m* nodes. We put an edge between nodes *i* and *j* if *x*_*i*_ and *x*_*j*_ are “close." There are two variations:

- *ϵ*−neighborhoods (parameter *ϵ* ∈ *R*): nodes *i* and *j* are connected by an edge if ||*x*_*i*_−*x*_*j*_||^2^ < *ϵ* where the norm is the usual Euclidean norm in *R* ^*n*^.
- *K *nearest neighbors (parameter *k* ∈ *N*): nodes *i* and *j* are connected by an edge if *i* is among *k* nearest neighbors of *j* or *j* among *k* nearest neighbors of *i*.

(2) *Choosing the Weights*. Here, as well, we have two variations for weighting the edges. *W* is a sparse symmetric  *m* × *m*  matrix with *W*_*ij*_ having the weight of the edge joining vertices *i* and *j*, and 0 if there is no such edge.

(a) Heat kernel (Parameter *t* ∈ *R*): if nodes *i* and *j* are connected, put
  (5) $$\begin{array}{c} W_{ij}=e^{-{||x_{i}-x_{j}||}^{2}/t}. \end{array}$$
(b) Simple-mined (no parameter): *W*_*ij*_ = 1 if and only if vertices *i* and *j* are connected by an edge.

(3) *Eigenmaps*. Compute the eigenvectors and eigenvalues for the generalized eigenvector problem:

(6) $$\begin{array}{c} XLX^{T}a=\lambda XDD^{T}a, \end{array}$$

where *D* is a diagonal matrix whose entries are column (or row, since *W* is symmetric) sums of *W*, *D*_*ii*_ = ∑*jW*_*ji*_. *L* = *D* − *W* is the Laplacian matrix. The *i*th column of matrix *X* is *x*_*i*_.

Let the column vectors *a*_0_,…, *a*_*i*−1_ be the solutions of (6), ordered according to their eigenvlaues, *λ*_0_ < ⋯<*λ*_*l*−1_. Thus, the embedding is as follows:

(7) $$\begin{array}{c} x_{i}⟶y_{i}=A^{T}x_{i},\;A=\left(a_{0},a_{1},\ldots ,a_{l-1}\right), \end{array}$$

where *y*_*i*_ is a *l*-dimensional vector and *A* is a *n × l *matrix.

In the proposed work, two types of features vectors are created (global and local). The global features extracted are based on wavelet and locality preserving projections (LPP). Two types of the wavelet functions are used (Daubechies (db2) and Symlets (sym2)). For each enhanced palm vein image, calculate the above functions of wavelet. The third levels for each earlier wavelet functions are computed and selected the approximation coefficients only as palm vein features. Then do the normalization for db2 and sym2 approximations coefficients based on *Z*-normalization. The two normalized feature vectors are fusions based on concatenation to create a new feature vector. The modern feature vector is projected based on locality preserving projections from high-dimension space to low-dimension space. That features vector is called wavelet locality preserving projections (WLPP).

**Figure 7:**
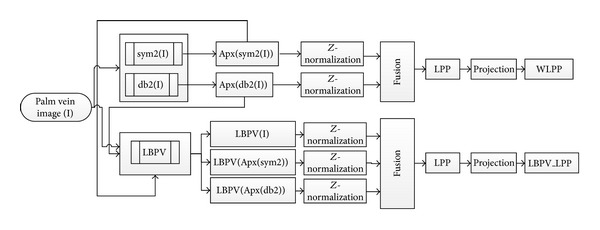
Flowchart of the feature vectors extraction.

The local features extracted based on local binary pattern variance (LBPV) and locality preserving projections (LPP). The LBPV is computed to the enhanced palm vein images and also to the approximation's coefficients of the wavelet functions Daubechies (db2) and Symlets (sym2). That process gave three feature vectors: one from enhanced image and the others from the wavelet approximation's coefficient. These feature vectors are normalized using *Z*-normalization. The normalization vectors are fused based on concatenation. Based on locality preserving projections project the concatenated feature's vector from high-dimension space to low-dimension space. That features vector is called local binary pattern variance_ locality preserving projections (LBPV_LPP).  Figure 7 shows the flowchart of creation the feature vectors.

## 4. Matching and Matching Score Fusion

The nearest neighbor method is used to compute the matching between the train set and test set. To measure the similarity between two biometric feature vectors, we used Euclidean distance as similarity measures. Let *y* denote the test feature vector and *x*_*i*_^*k*^ *i* = 1 ⋯ *C*_*k*_ *k* = 1 ⋯ *C* represent the *i*th gallery image of subject ID_*k*_, where *C*_*k*_ is the number of images of subject ID_*k*_ and *C* is the totally numbers of the images in the train set. The smallest Euclidean distance [24] is

(8) $$\begin{array}{c} {\text{ID}}_{y}=\arg\underset{k}{\min}{||y-x_{i}^{k}||}^{2}. \end{array}$$

In the proposed system, we have two feature vectors (WLPP and LBPV_LPP); by using the nearest neighbour compute the matching distance matrix to each features vector (WLPP distance matrix and LBPV_LPP distance matrix). In this paper, weighted sum fusion rule is applied to fusing the two matching distance matrices to obtain a final matching score to make a decision as shown in Figure 1. If the optimal weights of two modalities are searched exhaustively, it needs a twofold loop to reach to smallest value of EER.

## 5. Experimental Result and Conclusion 

### 5.1. Experimental Result

Experiments have been performed to evaluate the effectiveness of proposed palm vein verification methods based on multispectral palmprint PolyU database. The biometric research centre at the Hong Kong Polytechnic University has developed a real time multispectral palmprint capture device that can capture palmprint images under blue, green, red, and near-infrared (NIR) illuminations and has been used to construct a large-scale multispectral palmprint database. The database contains 6,000 images from 500 different palms for each of the above illumination. The proposed method uses the near-infrared (NIR) illumination images of PolyU multispectral palmprint database [25].

**Figure 8:**
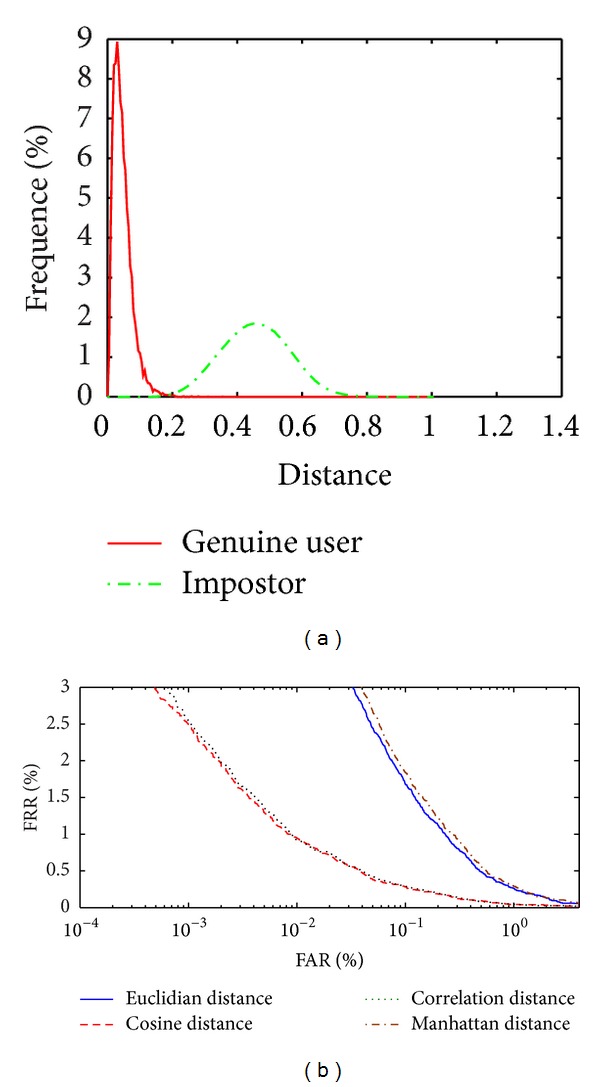
(a) Distribution of the genuine user and impostor without enhancement using cosine classifier. (b) ROC curve without enhancement to different classifiers.

Each individual has 12 palm vein images, six palm vein images are used for enrollment, and the other six images are used for the testing of each individual in the experiments. We used two performance measures, namely, the false rejection rate (FAR) and the false acceptance rate (FRR). For computing FRR value, we compare the biometric reference with all samples of the same individual. For computing FAR value, we compare the biometric reference of an individual with all samples from different individuals. The disparities distribution between intraclass and interclass matching result have been plotted. It shows the separation between genuine and impostors. The receiver operating characteristic (ROC) curve is between FRR and FAR. Equal error rate (EER) is the point where FRR is equal to FAR, and the smaller EER indicates a better performance. The nearest neighbour method is used to verify the feature vector from test set with the train set feature vectors and take the minimum distance for verification. The proposed system has many steps as image enhancement and the two types of features extraction methods and the matching classifier method. The following experimental results show when execute the system without image enhancement or without using dimension reduction or based on one type of feature extraction.  Figure 8(a) shows the distribution between genuine user and impostor to the smallest EER value classifier without image enhancement, Figure 8(b) shows the ROC curve when implement the system without image enhancement with different classifier, and Table 1 shows the EER value with different classifier methods.

**Figure 9:**
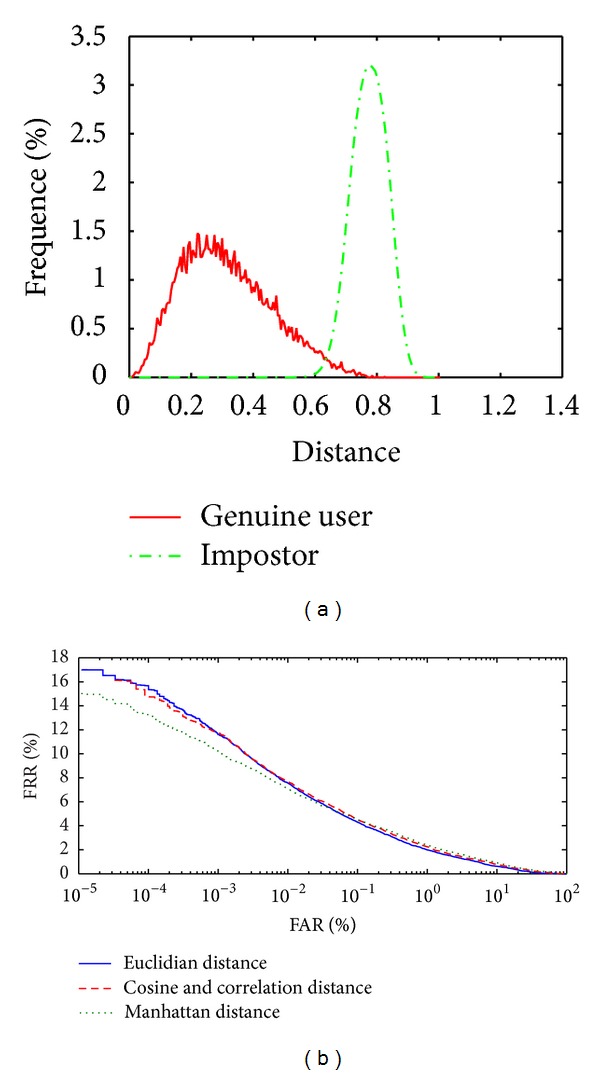
(a) Distribution of the genuine user and impostor without LPP using Euclidian classifier. (b) ROC curve without LPP to different classifiers.

Figures 8(a) and 8(b) and Table 1 show that the results without image enhancement are almost acceptable because the proposed system uses two types of feature extraction local and global (WLPP and LBPV_LPP) and also the fusion rule is applied to fuse the two matching distance matrices (WLPP distance matrix and LBPV_LPP distance matrix) to obtain a final matching score. We get the best result while using the Cosine matching classifier.  Figure 9(a) shows the distribution between genuine user and impostor to the smallest EER value classifier without LPP, Figure 9(b) shows the ROC curve when implement the system without LPP with different classifiers, and Table 2 shows the EER value with different classifier methods.

**Figure 10:**
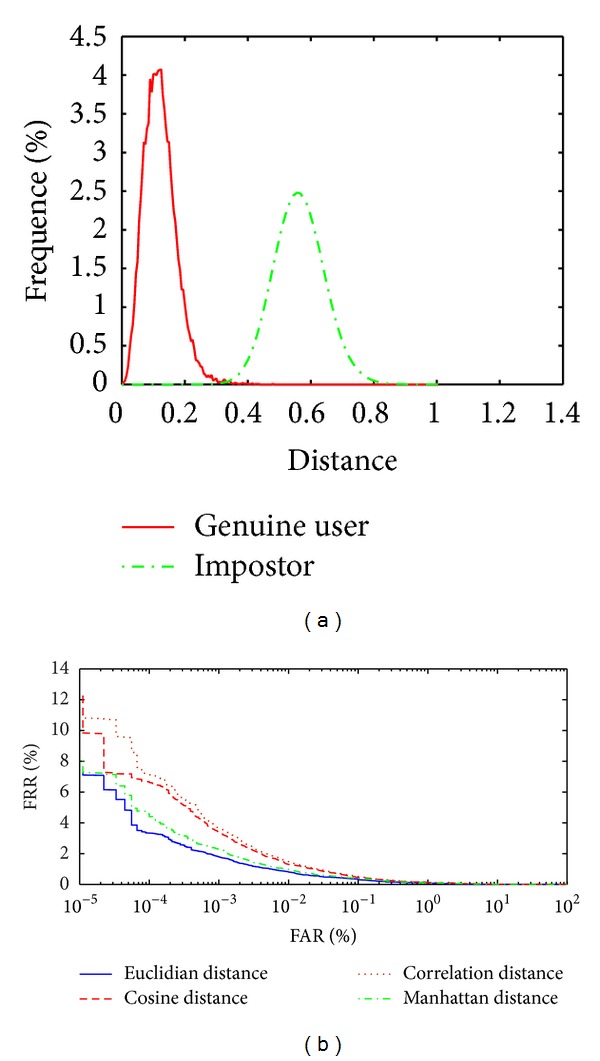
(a) Distribution of the genuine user and impostor based WLPP using Euclidian classifier. (b) ROC curve based WLPP to different classifiers.

Figures 9(a) and 9(b) and Table 2 show that the results without LPP are not acceptable because in the proposed system the feature extraction methods (Wavelet and LBPV) feature vectors have some redundancy values and applied to fusion of the two matching distance matrices (Wavelet distance matrix and LBPV distance matrix) to obtain a final matching score. We get the best result while using the Euclidian matching classifier.Figure 10(a) shows the distribution between genuine user and impostor to the smallest EER value classifier using WLPP, Figure 10(b) shows the ROC curve when implementing the system based on WLPP features vector with different classifier and Table 3 shows the EER value with different classifier methods.

**Figure 11:**
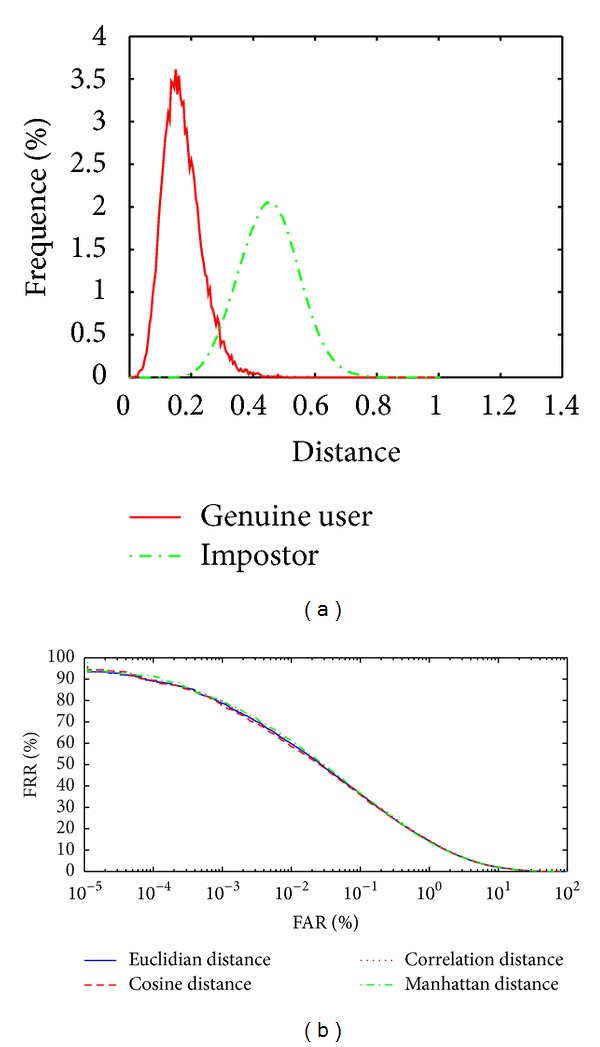
(a) Distribution of the genuine user and impostor based LBPV_LPP using Manhattan classifier. (b) ROC curve based LBPV_LPP to different classifiers.

Figures 10(a) and 10(b) and Table 3 show that the results based on WLPP features vector are almost acceptable because wavelet feature vector give useful information on the vein features as global feature vector and the LPP method remove all the redundancy features. Only wavelet distance matrix is used to obtain a final matching score. The best result is obtained while using the Euclidian matching classifier.  Figure 11(a) shows the distribution between genuine user and impostor to the smallest EER value classifier using LBPV_LPP, Figure 11(b) shows the ROC curve when implementing the system based on LBPV_LPP features vector with different classifier, and Table 4 shows the EER value with different classifier methods.

**Figure 12:**
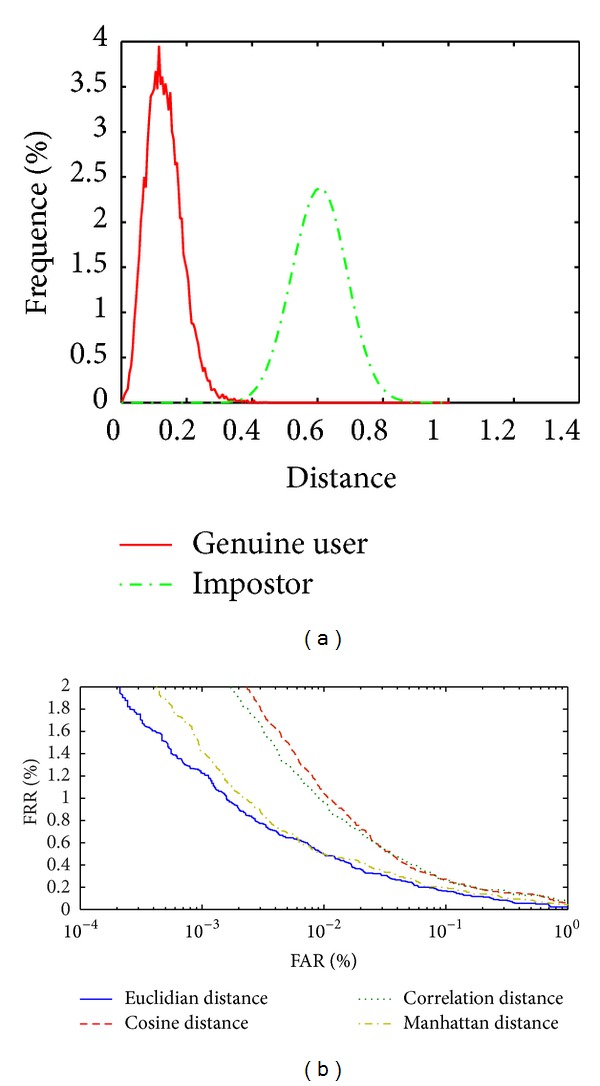
(a) Distribution of the genuine user and impostor to the proposed system using Euclidian classifier. (b) ROC curve to different classifiers.

Figures 11(a) and 11(b) and Table 4 show that the results based on LBPV_LPP are not acceptable because the LBPV_LPP get the local palm vein feature that is not enough for verification and only uses LBPV_LPP distance matrix to obtain a final matching score. We get the best result while using the Manhattan matching classifier.  Figure 12(a) shows the distribution between genuine user and impostor to the smallest EER value classifier for the proposed system.  Figure 12(b) shows the ROC curve when implementing the system based on image enhancement and WLPP and LBPV_LPP features vector with different classifiers and Table 5 shows the EER value with different classifier methods.

The distance distribution of genuine and impostor of the palm vein images is shown in Figure 12(a) and the ROC curve in Figure 12(b). The EER to the proposed system is 0.1378% by using nearest neighbor (Euclidian distance) method. This result is more acceptable from the other above experimental results because the image enhancement can remove all noise and make the images vein clearer. WLPP extracted the global features to the vein images and when combined with LBPV_LPP local features give the best result in verification.

Five methods for palm vein authentication are proposed for comparison. In the [3, 9, 11] the researchers worked on the PolyU database and we implement the methods used in [8, 10] for testing on the PolyU database.  Table 6 shows the comparison of our method and all the above methods.

From the results illustrated in Table 6 and Figures 12(a) and 12(b), it is verified that the proposed method has better performance from the methods that are described in [3, 8–11] and the lowest EER value to the proposed method is 0.1378% by using the Euclidian distance. The main benefit of the proposed method is that which uses the combination of the local and global feature vectors (WLPP and LBPV_LPP) and uses the matching score fusion method that reaches to lowest EER value.

### 5.2. Conclusion

This paper has addressed the problems of palm vein segmentation and verification. The matching filter is exploited to extract palm vein pattern. Then global and local features are used (WLPP and LBPV_LPP). Finally, the palm vein verification was implemented using Euclidian distance classifier and then weighted sum fusion rule is applied to combine the two matching distance matrices. By the proposed method we get a lower EER value equal to 0.1378%. We can conclude that the preprocessing step is important because the palm vein images are noisy and unclear. In addition, one type of the features vector (local or global) features is not enough for verification and the LPP method removes all the redundancy in the features vector. The experimental result shows the best performance when enhancing the vein images and use a fusion of two types of the feature vectors. This process gives us an accurate and robust personal verification system.

## Figures and Tables

**Table 1:** Experimental result without image enhancement.

Matching method	EER
Euclidian matching	0.4899%
Manhattan matching	0.5274%
Cosine matching	0.1888%
Correlation matching	0.1944%

**Table 2:** Experimental result without LPP.

Matching method	EER
Euclidian matching	1.6471%
Manhattan matching	1.9150%
Cosine matching	1.7721%
Correlation matching	1.7721%

**Table 3:** Experimental result based WLPP features vector.

Matching method	EER
Euclidian matching	0.2047%
Manhattan matching	0.2388%
Cosine matching	0.2669%
Correlation matching	0.2833%

**Table 4:** Experimental result LBPV_LPP.

Matching method	EER
Euclidian matching	4.5563%
Manhattan matching	4.4828%
Cosine matching	4.5998%
Correlation matching	4.7043%

**Table 5:** Experimental result proposed system.

Matching method	EER
Euclidian matching	0.1378%
Manhattan matching	0.1554%
Cosine matching	0.1835%
Correlation matching	0.1887%

**Table 6:** The EER comparisons with other methods.

Method	EER
Zhang et al. [3]	0.3091%
Lee [8]	1.111%
Sun and Abdulla [9]	0.66%
Bu et al. [10]	0.1559%
Al-juboori et al. [11]	0.2335%
Proposed method using Euclidian distance	0.1378%
